# Authors’ Reply to: Variability in Large Language Models’ Responses to Medical Licensing and Certification Examinations

**DOI:** 10.2196/50336

**Published:** 2023-07-13

**Authors:** Aidan Gilson, Conrad W Safranek, Thomas Huang, Vimig Socrates, Ling Chi, Richard Andrew Taylor, David Chartash

**Affiliations:** 1 Section for Biomedical Informatics and Data Science Yale University School of Medicine New Haven, CT United States; 2 Department of Emergency Medicine Yale University School of Medicine New Haven, CT United States; 3 Program of Computational Biology and Bioinformatics Yale University New Haven, CT United States; 4 School of Medicine University College Dublin National University of Ireland, Dublin Dublin Ireland

**Keywords:** natural language processing, NLP, MedQA, generative pre-trained transformer, GPT, medical education, chatbot, artificial intelligence, AI, education technology, ChatGPT, conversational agent, machine learning, large language models, knowledge assessment

We thank Epstein and Dexter [1] for their close reading of our paper, “How Does ChatGPT Perform on the United States Medical Licensing Examination? The Implications of Large Language Models for Medical Education and Knowledge Assessment” [2]. In response to their comments, we present the following points for clarification:

- While search engines such as Bing (Microsoft Corp) and Google (Google LLC) have been noted to implement geographic tuning when presenting their information retrieval results, there is no evidence or documentation that the version of ChatGPT (OpenAI) used in our work similarly alters its output given the geolocation of the user or the device that is being used. Notably, however, the integration of ChatGPT into other online services, such as Bing or Snapchat (Snap Inc), has made the information provided to those services (eg, time zone or geolocation) available to ChatGPT [3].
- Additionally, although it may be true that (dialectic) grammatical differences in the English language result in variability that may mimic the variability of prompt engineering, there is no empirical evidence that this alters the performance of ChatGPT. Further examination of the correlation between prompt engineering methods and within-sentence grammatical tuning or variability may alleviate these concerns in future research.
- Although it is a medical knowledge–based examination, the American Board of Preventive Medicine Longitudinal Assessment Program pilot for clinical informatics is not equivalent to the USMLE (United States Medical Licensing Examination). ChatGPT’s performance on this maintenance of certification examination has been examined by Kumah-Crystal et al [4], and we defer to their assessment as a more apt comparator.
- While Epstein and Dexter [1] offer a comparison between ChatGPT 3.5, ChatGPT 4.0, and Google Bard, it is unclear as to how the three have been statistically compared in terms of sample size and answer quality beyond performance on multiple-choice questions. Bootstrapping responses appear to address an element of variability in large language model (LLM) responses; however, a more robust statistical comparison is warranted alongside a comparison of nonbinarized LLM output performance.
- While there is no doubt that there is variability in the responses of LLMs to identical inputs (as these tools are nondeterministic in character), we do not believe this devalues the statistical significance or the quantitative validity of our results. As we are evaluating the performance of ChatGPT in the same situation as a student examinee, a single response is more applicable. Additionally, since we used a large sample size of questions, which accounted for model variability, we elected not to repeat questions multiple times.

## Abbreviations

LLM: large language model
USMLE: United States Medical Licensing Examination
